# Comparative Effects of Different Proteases on the Non-Volatile Taste Compounds and Volatile Flavor Fingerprints of *Sarcodon imbricatus* Hydrolysates

**DOI:** 10.3390/foods15111924

**Published:** 2026-05-29

**Authors:** Ju Guan, Yanlin Li, Tianyang Wang, Haibin Yuan, Hongfeng Jia, Wenjiao Fan, Wei Chen, Huachang Wu, Minglu Lan, Yuwen Yi

**Affiliations:** 1 Cuisine Science Key Laboratory of Sichuan Province, Sichuan Tourism University, Chengdu 610100, China; 2 College of Food and Biological Engineering, Chengdu University, Chengdu 610106, China; 3 Shenzhen Academy of Metrology and Quality Inspection, Shenzhen 518000, China; 4 Sichuan Cuisine Industrialization Engineering Research Center of Sichuan Higher Education Institutions, Sichuan Tourism University, Chengdu 610100, China

**Keywords:** *Sarcodon imbricatus*, enzymatic hydrolysis, free amino acids, 5′-nucleotides, HS-GC-IMS

## Abstract

Enzymatic hydrolysis with proteases efficiently releases taste active and volatile compounds from mushroom matrices; however, how protease specificity impacts the flavor characteristics of *Sarcodon imbricatus* (*S. imbricatus*) remains unclear. In this study, *S. imbricatus* was treated with Flavourzyme, Protamex, and Bromelain to produce hydrolysates, and the resulting changes in non-volatile taste-active compounds and volatile flavor characteristics were systematically evaluated by free amino acid analysis, 5′-nucleotides determination, electronic tongue, and HS-GC-IMS. The results show that enzymatic hydrolysis significantly affects its flavor characteristics. Flavourzyme treatment resulted in the highest degree of hydrolysis (29.24 ± 0.65%) and free amino acid (47.46 ± 1.02 mg/g) accumulation, whereas Protamex produced the highest equivalent umami concentration (EUC) value (115.81 g MSG/100 g), indicating stronger umami synergism between amino acids and 5′-nucleotides. Bromelain also altered the flavor characteristics after hydrolysis, but to a lesser extent. Electronic tongue analysis further showed that enzymatic hydrolysis generally enhanced umami and reduced bitterness, with Flavourzyme exhibiting the most pronounced effect. Meanwhile, HS-GC-IMS identified 69 volatile compounds and showed that FSIH and PSIH had more pronounced effects on the volatile profiles of *S. imbricatus*. Furthermore, 16 discriminatory markers (VIP > 1) were screened, indicating distinct volatile modulation by different proteases. Overall, Flavourzyme exhibited the most balanced performance and was considered the preferable protease for *S. imbricatus* hydrolysis under the present conditions. These findings provide a theoretical basis for its further processing and natural seasoning development.

## 1. Introduction

Edible mushrooms are widely recognized not only for their nutritional value but also for their distinctive flavor profiles, which have attracted increasing attention in the food industry [1,2]. Among the approximately 14,000 known mushroom species worldwide, about 3000 are considered edible, and China is a major producer and consumer [3]. *Sarcodon imbricatus* (*S. imbricatus*) is an edible wild tooth mushroom belonging to the family Bankeraceae and is widely distributed in East Asia, Europe, and North America. It is regarded as an “aromatic mushroom” due to its strong aroma [4]. The edible mushroom fruiting body is rich in bioactive constituents, including polysaccharides, adenosine, and triterpenes, which have been reported to exhibit antibacterial, anti-inflammatory, antioxidant, cholesterol-lowering, and immunomodulatory activities. Moreover, the fruiting body is stout and fleshy, with a delicate texture, and is also rich in nutrients such as proteins, minerals, and amino acids [5,6,7]. Edible mushrooms, with their unique flavor characteristics and excellent edible quality, can be regarded as high-quality raw materials for the production of natural seasonings. However, current studies on *S. imbricatus* have mainly focused on its bioactive constituents, whereas research on the extraction and utilization of its flavor compounds remains relatively limited.

Non-volatile taste compounds are an important material basis for taste perception in edible mushrooms, and among them, umami-related components are particularly important to their sensory quality [1]. Umami, recognized as the fifth basic taste, is regarded as one of the most representative taste attributes of edible mushrooms and is mainly derived from low-molecular-weight water-soluble compounds. Including umami amino acids such as glutamic acid and aspartic acid, as well as flavor 5′-nucleotides such as 5′-guanosine monophosphate (5′-GMP) and 5′-inosine monophosphate (5′-IMP); meanwhile, 5′-adenosine monophosphate (5′-AMP) may also contribute to umami formation to a certain extent [8,9]. However, mushroom proteins generally occur as intact macromolecular proteins and therefore make only a limited direct contribution to taste perception [10].

In this context, protease hydrolysis has been widely regarded as an effective approach for releasing taste-active compounds from edible mushrooms. Owing to its mild and controllable reaction conditions and high substrate specificity, it can convert these macromolecules into low-molecular-weight taste substances, such as free amino acids (FAAs) and peptides, thereby improving the taste characteristics of the resulting hydrolysates [11]. Nevertheless, the type of protease plays a decisive role in determining the hydrolysis performance and flavor quality of mushroom hydrolysates. Comparative studies based on different protease treatments have shown that, owing to differences in substrate specificity and preferred peptide-bond hydrolysis sites, proteases may vary substantially in their ability to promote protein degradation and release flavor precursors, thereby leading to distinct sensory characteristics. For example, Flavourzyme showed superior performance in improving protein degradation and flavor quality in *Lentinus edodes* hydrolysates [12], whereas Protamex treatment yielded the highest umami sensory score and EUC in *Agrocybe aegerita* hydrolysates [13]. Bromelain-derived *Pleurotus ostreatus* hydrolysate has also been reported to exhibit considerable potential as a natural flavor enhancer [14]. Therefore, a systematic comparison of different proteases is necessary to optimize the flavor quality of mushroom hydrolysates.

The flavor of edible mushrooms is jointly shaped by non-volatile taste compounds and volatile aroma compounds, with volatile organic compounds (VOCs) making important contributions to their overall flavor quality [15]. Headspace-gas chromatography-ion mobility spectrometry (HS-GC-IMS) has emerged as a powerful tool for volatile fingerprinting due to its high sensitivity, rapid response, and ability to distinguish isomers [16]. This technique has been successfully applied to characterize VOCs in various mushrooms, including *Tricholoma matsutake* and *Lentinus edodes* [17,18]. However, there has been no systematic study on the influence of the volatile fingerprint pattern of *S. imbricatus*.

The present study aimed to systematically investigate the effects of three different proteases (Flavourzyme, Protamex, and Bromelain) on the flavor characteristics of *S. imbricatus* hydrolysates (SIHs). The degree of hydrolysis, free amino acid composition, 5′-nucleotides content, and equivalent umami concentration (EUC) were measured, and the non-volatile taste profiles were evaluated using an electronic tongue. In addition, HS-GC-IMS combined with multivariate statistical analysis was employed to characterize the volatile fingerprints and identify key differential volatile markers. The results provide a theoretical basis for selecting appropriate proteases for the production of *S. imbricatus*-based natural seasonings and functional food ingredients.

## 2. Materials and Methods

### 2.1. Materials

*S. imbricatus* was purchased from a local agricultural market in Jiulong County, Ganzi Prefecture, Sichuan Province, China. The samples were vacuum-dried at 20 Pa and −50 °C until the moisture content was below 10% (fresh weight basis), ground into powder using a grinder, passed through a 60-mesh sieve, and stored in sealed containers in a cool, dry place. Flavourzyme (20 U/mg), Protamex (120 U/mg), and Bromelain (300 U/mg) were supplied by Shanghai Yuanye Bio-Technology Co., Ltd. (Shanghai, China). All chemical reagents and standard compounds were obtained from Shanghai Aladdin Biochemical Technology Co., Ltd. (Shanghai, China) and were of analytical grade.

### 2.2. Preparation of SIHs

*S. imbricatus* powder was suspended in deionized water at a material-to-liquid ratio of 1:20 (*w*/*v*) in 250 mL conical flasks and mixed using a magnetic stirrer. The pH of the suspension was adjusted to the optimal value for each enzyme using 1 M HCl or 1 M NaOH. According to the manufacturer’s specifications, the pH values were 7.5 for Flavourzyme, 7.5 for Protamex, and 7.0 for Bromelain. Adjusted to achieve a final enzyme-to-substrate ratio (E/S) of 4000 U/g. According to the enzyme systems employed, the hydrolysates prepared using Flavourzyme, Bromelain, and Protamex were designated as FSIH, BSIH, and PSIH, respectively. All experimental samples were placed in a 50 °C water bath for 2 h, with intermittent stirring every 10 min during hydrolysis. After the reaction, the samples were heated in a 90–100 °C water bath for 10 min to inactivate the enzymes. The hydrolysates were centrifuged at 5000 rpm and 4 °C, and the supernatants were collected as SIHs. The SIHs were stored at 4 °C for short-term storage before analysis, whereas samples intended for long-term storage were preserved at −60 °C. The control group was prepared under the same conditions, except that no protease was added. All samples were analyzed in triplicate.

### 2.3. Degree of Hydrolysis Assay

Total nitrogen content was determined using an automatic Kjeldahl nitrogen analyzer. The α-amino nitrogen content was determined using the formaldehyde titration method. The DH was calculated as the ratio of α-amino nitrogen to total nitrogen [13].

(1) $$\mathrm{DH}=\frac{m}{m_{1}}\;\times \;100\%$$

Among them, *m* represents the mass of α-amino nitrogen in the supernatant after enzymatic hydrolysis, and *m*_1_ represents the total nitrogen content before enzymatic hydrolysis.

### 2.4. Free Amino Acid Content

Free amino acids (FAAs) in SIHs were determined according to the method reported by Cai et al. [19], using an automatic amino acid analyzer (SYKAM, Munich, Germany) equipped with an LCA K07/Li column (150 mm × 4.6 mm). For amino acid analysis, aliquots (1 mL) of each sample were mixed with 9 mL of 7% (*w*/*v*) sulfosalicylic acid, followed by ultrasonication for 30 min to precipitate residual proteins and remove interfering components. The resulting solutions were filtered through 0.22 μm membrane filters prior to analysis. Chromatographic conditions were as follows: an injection volume of 50 μL, a reactor temperature of 130 °C, and a column temperature programmed from 38 to 74 °C. The ninhydrin reagent was delivered at a flow rate of 0.25 mL/min, and the total run time was 108 min.

### 2.5. Taste Activity Value (TAV)

TAV is an important indicator for evaluating the sensory contribution of taste compounds and is defined as the ratio of the actual concentration of a compound in the sample to its corresponding taste threshold [20]. Generally, compounds with a TAV > 1 can be perceived by humans and contribute significantly to the overall taste, whereas those with TAV < 1 make a relatively weak contribution. The TAV value is calculated as follows:

(2) $$\mathrm{TAV}\;=C_{1}/C_{2}$$

where *C*_1_ represents the concentration of the compound (mg/100 g) and *C*_2_ denotes its taste threshold (mg/100 g).

### 2.6. 5′-Nucleotides Content

The content of 5′-nucleotides was determined according to a previously reported method with minor modifications [21]. The HPLC system was equipped with an ultraviolet detector and a Venusil XBP C18 column (4.6 mm × 250 mm, 5 μm). The mobile phase consisted of methanol and 0.05 mol/L KH_2_PO_4_ solution (pH 4.5) at a volume ratio of 5:95, delivered at a flow rate of 1.0 mL/min, with UV detection at 254 nm. The column temperature was maintained at 25 °C. Each 5′-nucleotides was quantified by comparing its peak area with the corresponding calibration curve obtained from standard compounds. All samples were analyzed in triplicate.

### 2.7. EUC Assay

The EUC was expressed as g monosodium glutamate (MSG)/100 g, which was employed to assess the equivalent umami intensity contributed by the synergistic interaction of umami amino acids and 5′-nucleotides. A higher EUC value indicates a stronger overall umami perception [21]. The calculation was performed as follows:

(3) $$\mathrm{EUC}\;(g\;\mathrm{MSG}/100\;g)=\sum a_{i}b_{i}+1218\left(\sum a_{i}b_{i}\right)\left(\sum a_{j}b_{j}\right)$$

where *a_i_* and *b_i_* are the concentrations of umami amino acids (Aspartic acid or Glutamic acid) and umami nucleotides (5′-AMP, 5′-GMP and 5′-IMP), respectively; *a_j_* and *b_j_* are their corresponding relative umami coefficients (Glutamic acid = 1; Aspartic acid = 0.077; 5′-AMP = 0.18; 5′-GMP = 2.3 and 5′-IMP = 1) and 1218 is the synergistic constant.

### 2.8. Electronic Tongue Analysis

To characterize the taste-related profiles of SIHs, electronic tongue analysis was performed using an α-ASTREE system (Alpha MOS, Toulouse, France), equipped with five taste sensors for the detection of sourness (AHS), saltiness (CTS), umami (NMS), bitterness (SCS), and sweetness [22]. Electronic tongue measurements, 10 mL of each hydrolysate was diluted with 90 mL of deionized water, and an 80 mL aliquot of the diluted solution was transferred to a dedicated beaker. Data acquisition was conducted for 120 s with a sampling interval of 1.0 s and a stirring speed of 1 r/s. Each sample was measured ten times, and the average of three stable measurements was used as the final result.

### 2.9. HS-GC–IMS Analysis of Volatile Composition

The VOCs in the SIHs samples were analyzed using HS-GC-IMS (FlavourSpec, Gesellschaft für Analytische Sensorsysteme GmbH, Dortmund, Germany). GC–IMS analysis was carried out according to a previously reported method with minor modifications [23]. An automatic headspace sampling device coupled to a GC–IMS system was used for VOC analysis. Separation was performed on a WAX column (30 m × 0.53 mm; RESTEK, Bellefonte, PA, USA). Briefly, 5 mL of the sample was transferred into a 20 mL headspace vial and incubated at 60 °C for 20 min. After incubation, 500 μL of headspace gas was injected in splitless mode at 80 °C, using nitrogen (99.99% purity) as the carrier gas. The carrier gas was programmed as follows: 2 mL/min for 2 min, 30 mL/min for 8 min, 100 mL/min for 10 min, and 150 mL/min for 5 min. Retention indices (RI) were calculated using a series of C4–C9 n-ketones as external standards. Volatile compounds were tentatively identified by comparing their RI and drift time (DT) with those in the GC–IMS library.

The relative odor activity value (ROAV) was calculated according to a previously reported method [24]. The compound with the highest odor activity value was assigned an ROAV of 100, and the ROAV of each volatile compound was calculated as follows:

(4) ROAV=100×CiTi∕CmaxTmax

where *C_i_* and *T_i_* represent the relative content and odor threshold of compound i, respectively, and *C_max_* and *T_max_* represent the relative content and odor threshold of the compound with the highest odor activity value.

### 2.10. Statistical Analyses

Differences among samples were statistically analyzed using IBM SPSS Statistics 26 (IBM, Armonk, NY, USA), and significance was defined at *p* < 0.05. Data are expressed as mean ± standard deviation. Orthogonal partial least squares-discriminant analysis (OPLS-DA) was performed using SIMCA 14.1 (Umetrics, Umea, Sweden) to distinguish the different treatment groups and identify the variables contributing to sample separation. Heatmaps and other graphical visualizations were produced using Origin 2021 (OriginLab Corporation, Northampton, MA, USA) and ChiPlot. Two OPLS-DA models were established based on free amino acid and 5′-nucleotides data, and HS-GC-IMS volatile compound data, respectively. In the models, X represented the measured variable matrix, whereas Y represented the sample classes, including CK, FSIH, PSIH, and BSIH. Model performance was evaluated using R^2^X, R^2^Y, and Q^2^, which indicate the explanatory ability for the X variables, goodness of fit for class discrimination, and predictive ability of the model, respectively. A 200-times permutation test was used to evaluate potential overfitting, and variables with variable importance in projection (VIP) values greater than 1 were considered important discriminant markers.

## 3. Results and Discussion

### 3.1. Enzyme Effects on Degree Hydrolysis (DH)

DH represented the extent of peptide bond hydrolysis during protein hydrolysis and was commonly used as a key indicator of enzymatic hydrolysis performance, reflecting, to a certain extent, the efficiency of protein hydrolysis [21]. Accordingly, DH was employed in this study as the evaluation index to compare the hydrolytic effect on the DH of the SIHs. Before enzymatic hydrolysis, the total nitrogen content of *S. imbricatus* was 28.75 g/kg. As shown in Table 1, compared with the CK group (9.35%), all protease-treated groups showed significantly higher DH values, ranging from 17.28% to 29.24% (*p* < 0.05), indicating that protease treatment markedly promoted the hydrolysis of *S. imbricatus* proteins.

Among the enzymatic hydrolysis groups, FSIH showed the highest DH value (29.24%), which may be attributed to the combined endopeptidase activity and strong exopeptidase activity of Flavourzyme [6]. The endopeptidase activity helps to disrupt the secondary and tertiary structures of proteins, while the exopeptidase activity can sequentially cleave peptide bonds from the protein termini, resulting in more extensive protein degradation and the production of a large amount of small peptides and FAAs, thereby generating the highest DH value [25]. However, Bromelain and Protamex are both mainly endopeptidases that preferentially hydrolyze internal peptide bonds, thereby showing limited ability to further degrade peptides into smaller fragments or FAAs, which ultimately leads to lower DH values [22]. It is noteworthy that, although Protamex acts predominantly as an endopeptidase, its additional exopeptidase activity may contribute to a relatively higher DH than that observed for Bromelain.

### 3.2. Effects of Enzymolysis on the Free Amino Acid Analysis

**Figure 1:**
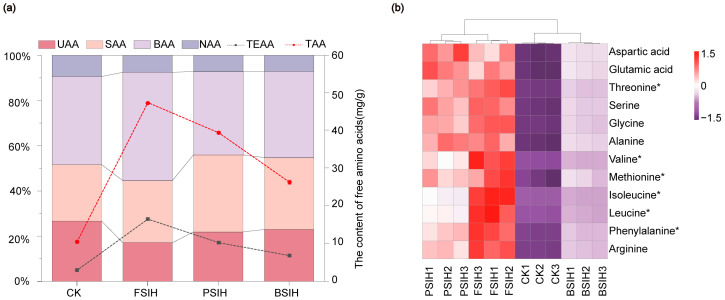
Composition characteristics and TAV distribution of free amino acids under different enzymatic hydrolysis treatments. (**a**) Relative proportions of taste-active amino acids and contents of TAA and TEAA (**b**) Heatmap of TAVs for free amino acids (“*” indicates essential amino acids).

FAAs are important non-volatile taste compounds in edible mushrooms and contribute substantially to umami perception [1]. In this study, the FAA content in the CK and three enzyme-treated samples was measured. A total of 17 FAAs, including seven essential amino acids. According to their taste characteristics, these amino acids were classified into umami amino acids (UAA), sweet amino acids (SAA), bitter amino acids (BAA), and non-taste amino acids (NAA), as shown in Table 1. Enzymatic hydrolysis significantly increased both total amino acids (TAA) and total essential amino acids (TEAA) (Figure 1a). Protease treatment significantly increased the contents of TAA and TEAA from 10.60 and 3.06 mg/g in CK to 26.35–47.46 and 6.92–16.62 mg/g, respectively (*p* < 0.05), indicating that enzymatic hydrolysis promoted protein breakdown and facilitated the release of FAAs. The elevated TEAA content further suggested an improvement in the nutritional quality of the hydrolysates [26]. Notably, this effect was most pronounced in FSIH [13]. The strong exopeptidase activity of Flavourzyme promoted further protein degradation and thereby favored the release of FAAs, in agreement with its higher DH value. In addition to increasing the overall FAA content, enzymatic hydrolysis markedly changed the relative proportions of different amino acid classes. BAA was the predominant amino acid fraction in all groups, with the highest proportion observed in FSIH, whereas SAA showed relatively higher proportions in PSIH and BSIH. In contrast, UAA did not show a proportional increase after hydrolysis, which was likely due to a dilution effect associated with the marked increase in total FAA content. Therefore, the TAV of each FAA was calculated based on reported taste thresholds (Table 1).

Generally, compounds with higher TAVs contribute more strongly to overall taste perception. The detailed TAV values are listed in Table S1, and their distribution patterns were further visualized using a heatmap (Figure 1b). The results indicated that enzymatic hydrolysis enhanced the direct taste contribution of FAAs in SIHs. Glutamic acid remained far above the threshold in all samples, whereas aspartic acid increased from subthreshold levels in CK to TAV > 1 in all hydrolysates, indicating that protease treatment enhanced the direct umami contribution of FAAs in SIHs. Within the SAA fraction, alanine consistently exhibited TAV > 1 in all groups, whereas serine exceeded the threshold only in FSIH and PSIH, suggesting a moderate increase in sweetness-related contribution. BAAs were the predominant amino acid group in all samples. Valine and arginine were the main bitter-contributing amino acids, while isoleucine, leucine, and phenylalanine exceeded the threshold only after enzymatic hydrolysis. Taken together, enzymatic hydrolysis increased the taste contribution of umami FAAs and partially enhanced that of sweet FAAs in SIHs. Although several bitter FAAs also showed increased potential contributions after hydrolysis, their influence on overall bitterness may be limited because bitter peptides generated during hydrolysis are also important contributors to bitterness [27].

### 3.3. Effects of Enzymolysis on the 5′-Nucleotides, and EUC Analysis

In addition to FAAs, 5′-nucleotides are also important non-volatile taste compounds in edible mushrooms. Some nucleotides possess intrinsic umami properties and can also act synergistically with amino acids to improve food flavor. In SIHs, 5′-uracil monophosphate (5′-UMP) and 5′-GMP were the predominant nucleotide species. As shown in Table 2, the total nucleotide content in the hydrolysates was significantly higher than that in the CK group (*p* < 0.05), suggesting that enzymatic hydrolysis promoted cell wall disruption and thereby facilitated nucleotide release. In addition, protease treatment markedly altered the nucleotide profile in an enzyme-dependent manner, with FSIH showing the highest total 5′-nucleotides content, followed by PSIH, likely due to differences in protease hydrolysis patterns that affected nucleotide release [28]. From the perspective of taste contribution, 5′-GMP, 5′-IMP, and 5′-AMP are more directly involved in the synergistic enhancement of umami with glutamic acid. Notably, 5′-AMP also imparts a certain sweetness, which may help alleviate the bitterness generated during mushroom hydrolysis [21]. In contrast, 5′-UMP exhibits only a weak enhancing effect, whereas 5′-cytidine monophosphate (5′-CMP) contributes little to the overall taste profile [29]. In the present study, 5′-GMP was the major umami-related nucleotide in SIHs and was significantly increased after enzymatic hydrolysis, suggesting that protease treatment, particularly Protamex, favored the retention or release of key umami nucleotides [30].

The taste profile of the samples depended on the combined effects of multiple taste-active compounds rather than on any single component alone [31]. Because umami amino acids and 5′-nucleotides can act synergistically, the EUC was calculated to estimate the umami potential of the samples. Compared with the enzymatic hydrolysates (76.35–115.81 g MSG/100 g), the control group showed a significantly lower EUC value (27.73 g MSG/100 g), whereas PSIH exhibited the highest EUC [13]. These results indicate that enzymatic hydrolysis increased the potential umami intensity of *S. imbricatus*, although the extent of the increase differed among protease treatments.

### 3.4. Multivariate Statistical Analysis of Non-Volatile Flavor Compounds

**Figure 2:**
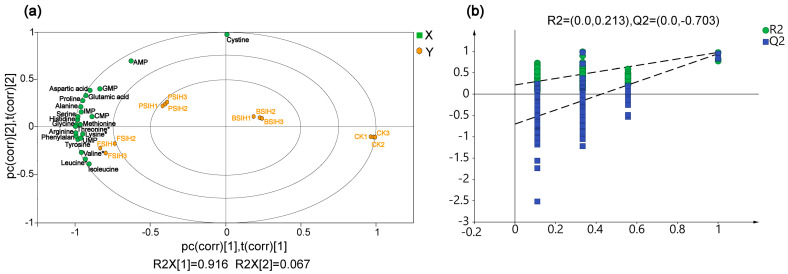
OPLS-DA of free amino acids and 5′-nucleotides in different enzymatic hydrolysates. (**a**) loadings plot; (**b**) 200-permutation test for model validation (The dashed lines represent the regression lines of R^2^ and Q^2^ values obtained from the 200 permutation tests, “*” indicates essential amino acids).

To better characterize differences in non-volatile taste-active compounds among hydrolysates produced by different proteases, an OPLS-DA model was established based on FAAs and nucleotides. As shown in Figure 2a, the first and second components explained 91.6% and 6.7% of the X variation, respectively, indicating that the model captured most of the variation in the dataset. CK was clearly separated from all enzymatic hydrolysates along the primary discriminant direction. In contrast, most FAAs and nucleotides were distributed on the opposite side, suggesting that proteolysis promoted the release of non-volatile taste compounds and markedly altered the taste profile of SIHs. Among the hydrolysates, PSIH was located closer to 5′-AMP, 5′-GMP, glutamic acid, aspartic acid, and proline, indicating a stronger association with nucleotide-related umami enhancement and several umami- or sweetness-related amino acids. FSIH, by contrast, was positioned nearer to leucine, isoleucine, valine, and phenylalanine, suggesting that its non-volatile taste profile was mainly shaped by a distinct FAA release pattern. BSIH was located between CK and the other hydrolysates and showed a relatively weaker association with most FAAs and 5′-nucleotides, indicating that Bromelain induced comparatively mild changes in non-volatile composition. Furthermore, the 200-times permutation test gave an R^2^ intercept of 0.213 and a negative Q^2^ intercept of −0.703, confirming that the model was robust and not overfitted (Figure 2b). Overall, these results indicated that different proteases shaped the differentiated non-volatile taste characteristics of SIHs by inducing distinct FAA and nucleotide release patterns. This was generally consistent with the compositional results described above.

### 3.5. E-Tongue Analysis

**Figure 3:**
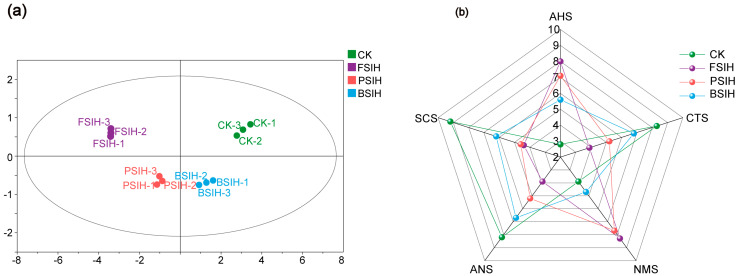
Effects of different enzymatic hydrolysates on electronic tongue response characteristics. (**a**) Principal component analysis (PCA) score plot; (**b**) radar plot of responses from all electronic tongue sensors.

The electronic tongue, as a biomimetic taste sensing technique, can rapidly and objectively characterize the overall taste attributes of liquid samples, thereby reducing the subjective bias associated with traditional sensory evaluation while offering good stability and sensitivity [32]. In the present study, the electronic tongue results demonstrated that different protease treatments markedly altered the overall taste profile of SIHs. As shown in the radar plot (Figure 3b), all three enzymatic hydrolysates generally exhibited stronger umami responses and lower bitterness responses than CK, indicating that proteolysis did not lead to obvious bitter taste accumulation but instead contributed to a more coordinated non-volatile taste profile [33].

Among the three hydrolysates, FSIH exhibited the strongest umami response, followed by PSIH, whereas BSIH showed a relatively weaker umami-enhancing effect. Both FSIH and PSIH were characterized by lower bitterness and higher sourness, accompanied by reduced sweetness and saltiness. In contrast, BSIH showed more moderate changes across the taste attributes and remained at an intermediate level overall. This trend was generally consistent with the FAA, 5′-nucleotides, and EUC analyses, indicating that the electronic tongue responses were closely associated with changes in non-volatile taste compounds. Notably, the umami response detected by the electronic tongue did not fully parallel the EUC values, suggesting that the overall umami expression of the samples was not determined solely by FAAs and taste-active nucleotides. Other non-volatile compounds, particularly low-molecular-weight taste-active peptides, may also have contributed to the final taste perception [34].

To further discriminate the overall taste differences among the samples, principal component analysis (PCA) was performed based on the electronic tongue responses (Figure 3a). The first two principal components, PC1 and PC2, accounted for 92.7% and 6.9% of the total variance, respectively, with a cumulative contribution of 99.6%, indicating that these two components were sufficient to describe the overall taste variation among the samples. In the PCA score plot, CK was clearly separated from the three hydrolysate groups, while FSIH, PSIH, and BSIH were also distinctly distributed along PC1. These results further confirmed that protease type was a key factor governing the final taste characteristics of SIHs, and that the observed taste differences arose from the combined variation in multiple taste dimensions rather than from a single taste attribute alone.

### 3.6. HS-GC-IMS Analysis

**Figure 4:**
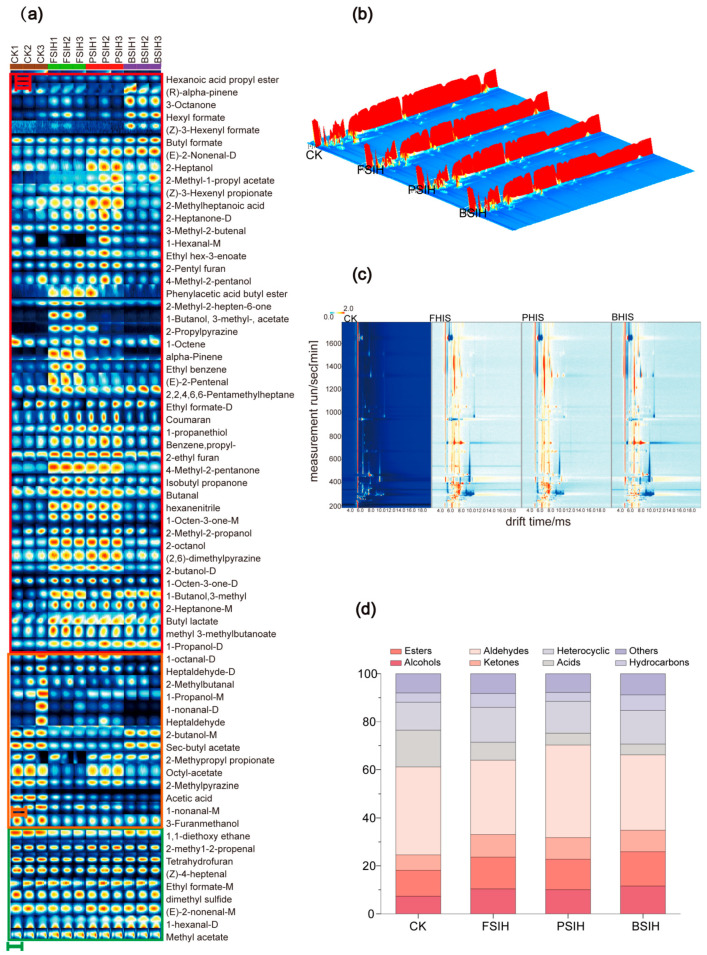
HS-GC-IMS characterization of VOCs in SIHs under different enzymatic hydrolysis treatments. (**a**) GC-IMS fingerprint of volatile compounds identified in SIHs. (**b**) Three-dimensional topographic plots of volatile compounds in SIHs. (**c**) Comparison of volatile compound spectra in SIHs based on colorized difference plots. (**d**) Relative proportions of different volatile compound classes calculated from VOC peak intensities.

Given its high sensitivity and rapid response, HS–GC–IMS has been extensively utilized in mushroom flavor research [35]. Accordingly, it was applied here to characterize the volatile profile of *S. imbricatus* subjected to different protease treatments (Figure 4). As shown in Figure 4b,c, clear differences in volatile profiles were observed between the protease-treated samples and the CK group. A three-dimensional topographic map showed noticeable variations in peak intensity and signal distribution among samples, indicating that protease treatment markedly altered the abundance and composition of volatile compounds. Two-dimensional topographic difference map, red and blue regions indicate signal intensities higher and lower than those of CK, respectively. The predominance of red signals in the enzyme-treated samples suggested that enzymatic hydrolysis generally promoted the accumulation of volatile compounds, while the variation in color intensity and distribution among treatments further reflected protease-dependent differences in volatile enrichment.

A total of 69 VOCs, including both monomeric (M) and dimeric (N) forms, were identified in the enzymatic hydrolysates of *S. imbricatus* (Table S2). To further clarify VOC composition, the identified compounds were classified into alcohols, esters, ketones, aldehydes, acids, heterocyclic compounds, hydrocarbons, and others. Their relative proportions were calculated by normalizing the summed peak volume of each class to the total VOC peak volume (Figure 4d). Aldehydes were the dominant volatile class in all samples, accounting for 30.88–38.44% of the total VOC signals. Given their generally low odor thresholds, aldehydes may strongly influence the overall aroma profile and mainly contribute grassy, fatty, fruity, and oily notes. These compounds are mainly formed through lipid oxidation, particularly the oxidative degradation of unsaturated fatty acids, as well as amino acid degradation during processing. Linoleic acid is regarded as one of the major precursors of aldehydes and can be oxidized through the lipoxygenase–hydroperoxide lyase (LOX–HPL) pathway to generate straight-chain aldehydes such as nonanal, hexanal, (E)-2-nonenal, and heptanal [36], whereas some aldehydes may also arise from amino acid catabolism via transamination and decarboxylation reactions [37]. Protease treatment increased the proportions of alcohols, esters, ketones, and heterocyclic compounds, suggesting that enzymatic hydrolysis promoted the formation or release of several aroma-related volatile classes. Esters are generally formed through esterification between acids and alcohols and usually contribute sweet and fruity notes [38]. Among the heterocyclic volatiles, furans and pyrazines were the major groups detected. Pyrazines, an important class of nitrogen-containing volatiles, are commonly generated through Strecker degradation of amino acids and Maillard reactions during heating and are typically associated with roasted notes. By contrast, although furans are present in mushrooms, their contribution to the characteristic mushroom aroma is generally limited [2]. Ketones and alcohols were also widely detected and are mainly associated with lipid oxidation and amino acid metabolism during processing. Representative compounds identified in this study included 1-octen-3-one and 3-octanone, as well as 2-heptanol and 3-methyl-1-butanol, which are considered important contributors to the mushroom-like and fruity aroma characteristics of edible fungi [39,40]. In contrast, acids decreased from 15.29% in CK to 4.44–7.52% in the hydrolysates, suggesting that enzymatic hydrolysis reduced the relative contribution of acidic and pungent aroma notes.

The distribution patterns of the identified VOCs were visualized using an IMS fingerprint plot (Figure 4a) to facilitate comparison among different treatments. The resulting chromatographic fingerprints could be broadly divided into three regions. Region I comprised compounds detected in all four groups and exhibited generally stable signal intensities across treatments, indicating that these volatiles represented the relatively stable aroma profile of *S. imbricatus* and were less influenced by protease type. Region II included VOCs that showed relatively high signal intensities in the CK group but decreased markedly after enzymatic hydrolysis. These compounds were mainly aldehydes, alcohols, esters, and organic acids, including heptanal, octanal, nonanal, 1-propanol-M, 2-butanol-M, sec-butyl acetate, 2-methylpropyl propionate, octyl acetate, and acetic acid. Region III consisted of compounds whose signal intensities increased to different extents after hydrolysis, mainly low-molecular-weight alcohols, ketones, and some esters, such as 3-methyl-1-butanol, 2-heptanone, butyl lactate, and methyl 3-methylbutanoate. Notably, these changes differed among protease treatments. In the PSIH and FSIH, sulfur-containing compounds, pyrazines/furans, and several aldehydes and ketones associated with lipid oxidation showed more pronounced increases, including 1-propanethiol, 2,6-dimethylpyrazine, 2-ethylfuran, 1-octen-3-one, and butanal. By contrast, the BSIH showed relatively higher signal intensities for α-pinene, 3-octanone, hexyl formate, and (E)-2-nonenal. In edible mushrooms, C8 volatiles are recognized as important aroma-active compounds responsible for the characteristic mushroom-like odor [41]. In the present study, representative C8 compounds, including 1-octen-3-one, 3-octanone, 2-octanol, and 1-octanal, generally increased after enzymatic hydrolysis. Overall, these results indicate that enzymatic hydrolysis markedly reshaped the VOC profile of *S. imbricatus* and promoted the accumulation of compounds associated with its characteristic mushroom-like aroma.

### 3.7. Multivariate Analysis of VOCs of S. imbricatus Under Different Protease Treatments

**Figure 5:**
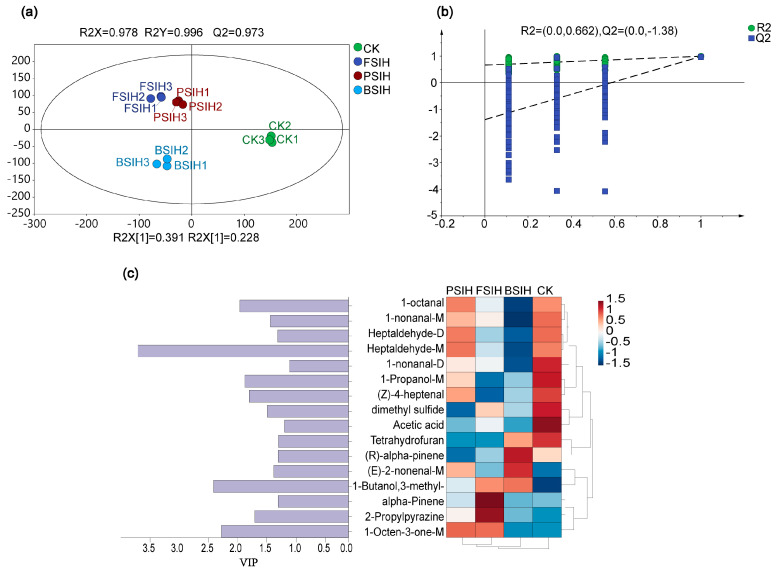
(**a**) Score plot of OPLS-DA; (**b**) Cross-substitution plot of 200 permutation tests; (**c**) VIP values and clustered heatmap of key differential volatile compounds based on GC-IMS.

To further characterize treatment-dependent differences in volatile compounds, an OPLS-DA model was established based on the HS-GC-IMS data. As shown in Figure 5a, the model showed excellent explanatory and predictive performance, with R^2^Y = 0.996 and Q^2^ = 0.973. CK was mainly distributed in the fourth quadrant, FSIH and BSIH in the second and third quadrants, respectively, and PSIH near the boundary between the first and second quadrants. As shown in Figure 5b, the 200-times permutation test yielded intercepts of R^2^ = 0.662 and Q^2^ = −1.38, and the negative Q^2^ intercept confirmed that the model was not overfitted. Compounds with VIP values > 1 were selected as discriminant volatile markers (Figure 5c). Sixteen compounds with VIP > 1 were identified. CK exhibited higher levels of 1-nonanal, 1-propanol, (Z)-4-heptenal, dimethyl sulfide, acetic acid, and tetrahydrofuran, which are primarily associated with endogenous lipid oxidation and metabolic processes in mushrooms [42]. After protease treatment, proteins were hydrolyzed into small peptides and FAAs, which serve as key precursors for flavor compound formation, leading to a shift in flavor formation pathways [43]. Protamex mainly exhibits endopeptidase activity with limited exopeptidase activity, resulting in partial structural disruption and a relatively low release of FAAs. Consequently, lipid oxidation-derived aldehydes such as 1-octanal, 1-nonanal, and heptanal decreased compared to CK, but to a lesser extent than in the other enzyme-treated groups. In contrast, Flavourzyme, characterized by dominant exopeptidase activity, extensively hydrolyzes peptides into FAAs, thereby intensifying Strecker degradation and Maillard reactions [44]. This favors the generation of key aroma compounds such as 3-methyl-1-butanol and 2-propylpyrazine. (E)-2-Nonenal, a typical oxidation product of unsaturated fatty acids, is closely associated with lipid exposure and oxidative processes [44]. It was most abundant in BSIH, which may be attributed to the proteolytic characteristics of Bromelain, as its endopeptidase activity is more likely to disrupt the protein matrix. Similarly, the elevated level of (R)-α-pinene may also arise from this structural disruption [24]. Notably, 1-octen-3-one-M showed higher signal intensities in FSIH and PSIH. As a characteristic C8 volatile associated with mushroom-like aroma, its increase may be related to the more pronounced mushroom-like notes of these two hydrolysates. These results suggested that the VIP > 1 compounds primarily acted as treatment-dependent discriminant markers and reflected the differential effects of proteases on volatile composition.

### 3.8. ROAV Analysis of Key Aroma Compounds in S. imbricatus

To further estimate the potential contribution of volatile compounds to the overall aroma profile, ROAV analysis was performed. In general, compounds with ROAV ≥ 1 are considered potential key aroma-active compounds, whereas those with 0.1 ≤ ROAV < 1 may influence the aroma profile through modifying or synergistic effects [24]. In the present study, compounds with ROAV values > 0.1 were further examined (Table S3). Among them, 1-octen-3-one showed an ROAV of 100 across all samples, indicating that it was the dominant aroma-active compound common to all groups and the principal contributor to the characteristic mushroom-like aroma [45]. In addition, (Z)-4-heptenal, dimethyl sulfide, and (E)-2-nonenal also showed relatively high ROAV values. In addition, (Z)-4-heptenal, dimethyl sulfide, and (E)-2-nonenal-D also showed relatively high ROAV values, indicating that they contributed to the aroma nuances of SIHs. Specifically, (Z)-4-heptenal mainly contributed dairy- and cream-like notes, which may provide a soft background aroma to the hydrolysates. Dimethyl sulfide was associated with cabbage-like and sulfurous notes, suggesting its contribution to the cooked vegetable-like and earthy aroma characteristics commonly found in edible fungi. (E)-2-nonenal-D mainly contributed cucumber-like, fatty, and green notes, reflecting lipid oxidation-related aroma characteristics. These compounds were not the primary source of the characteristic mushroom-like aroma, but they may have enriched the overall aroma profile of *S. imbricatus* hydrolysates by providing dairy-like, sulfurous, earthy, green, and fatty aroma nuances. In addition, nonanal and heptanal showed ROAV values within or close to the range of 0.1–1, suggesting that they were more likely to play modifying roles rather than act as major aroma contributors. This reflects a general feature of food aroma systems, in which only a few volatile compounds dominate the overall aroma, while others mainly contribute background or modifying notes [46].

## 4. Conclusions

This study investigated the effects of different proteases on the non-volatile taste-active compounds and volatile flavor characteristics of *S. imbricatus*. Enzymatic hydrolysis promoted the release of FAAs and 5′-nucleotides and improved the umami-related taste profile of the hydrolysates. Among the tested enzymes, Flavourzyme was more effective in promoting protein hydrolysis and amino acid accumulation, whereas Protamex showed a greater advantage in nucleotide release and yielded the highest EUC value (115.81 g MSG/100 g), indicating a stronger contribution to nucleotide-related umami synergism. Although Bromelain did not exhibit a distinct advantage in these chemical indices, it still improved the taste characteristics of the hydrolysates compared with the CK group. E-tongue analysis further revealed clear differences in the overall taste profiles among the protease-treated hydrolysates, indicating that enzymatic hydrolysis generally enhanced umami while reducing bitterness, with Flavourzyme showing the most pronounced effect in this regard. Different protease treatments markedly reshaped the volatile profile of *S. imbricatus*. HS-GC-IMS identified 69 volatile compounds, including several representative mushroom-like aroma compounds such as 1-octen-3-one, 2-heptanol, and 2-methyl-2-hepten-6-one. The overall fingerprint plots revealed marked differences in the distribution and intensity of volatile signals among treatments, with FSIH and PSIH showing denser and stronger responses, suggesting that these two treatments more effectively promoted the release and/or accumulation of volatile compounds. Moreover, 16 discriminatory markers (VIP > 1) were identified, further highlighting the distinct modulation of volatile characteristics by different proteases. ROAV analysis further identified four major aroma-active compounds and two additional aroma modifiers. Overall, Flavourzyme exhibited the most balanced performance among the tested proteases and was therefore considered the preferable choice for the hydrolysis of *S. imbricatus* under the present conditions. These findings provide a theoretical basis for the development of *S. imbricatus* as a natural seasoning ingredient or functional food components.

## Figures and Tables

**Table 1:** Degree of hydrolysis and free amino acid concentrations (mg/g) in SIHs.

	Taste Threshold (mg/100 g)	CK	FSIH	PSIH	BSIH
DH (%)		9.35 ± 0.54 ^d^	29.24 ± 0.65 ^a^	24.88 ± 0.51 ^b^	17.28 ± 0.47 ^c^
Umami					
Aspartic acid	100.00	0.74 ± 0.02 ^c^	2.03 ± 0.17 ^a^	2.33 ± 0.20 ^a^	1.56 ± 0.03 ^b^
Glutamic acid	30.00	2.09 ± 0.05 ^c^	6.09 ± 0.53 ^a^	6.71 ± 0.43 ^a^	4.50 ± 0.15 ^b^
Total		2.82 ± 0.07 ^c^	8.12 ± 0.51 ^a^	9.04 ± 0.41 ^a^	6.06 ± 0.17 ^b^
Sweet					
Threonine *	260.00	0.52 ± 0.01 ^d^	2.20 ± 0.09 ^a^	1.83 ± 0.12 ^b^	1.22 ± 0.03 ^c^
Serine	150.00	0.46 ± 0.02 ^c^	1.73 ± 0.09 ^a^	1.58 ± 0.13 ^a^	1.05 ± 0.02 ^b^
Glycine	130.00	0.14 ± 0.01 ^d^	0.61 ± 0.02 ^a^	0.51 ± 0.03 ^b^	0.34 ± 0.01 ^c^
Alanine	60.00	1.40 ± 0.04 ^c^	4.54 ± 0.32 ^a^	4.57 ± 0.31 ^a^	3.06 ± 0.07 ^b^
Proline	-	0.14 ± 0.01 ^d^	3.96 ± 0.20 ^b^	5.56 ± 0.18 ^a^	2.70 ± 0.05 ^c^
Total		2.65 ± 0.09 ^c^	13.03 ± 0.16 ^a^	14.05 ± 0.45 ^a^	8.37 ± 0.18 ^b^
Bitter					
Valine *	40.00	0.78 ± 0.03 ^d^	4.23 ± 0.29 ^a^	2.69 ± 0.19 ^b^	1.79 ± 0.04 ^c^
Methionine *	30.00	0.05 ± 0.01 ^c^	0.27 ± 0.04 ^a^	0.22 ± 0.02 ^a^	0.15 ± 0.01 ^b^
Isoleucine *	90.00	0.50 ± 0.01 ^d^	3.49 ± 0.16 ^a^	1.74 ± 0.11 ^b^	1.17 ± 0.03 ^c^
Leucine *	190.00	0.84 ± 0.03 ^d^	5.63 ± 0.54 ^a^	3.16 ± 0.20 ^b^	2.11 ± 0.04 ^c^
Phenylalanine *	90.00	0.70 ± 0.02 ^d^	3.55 ± 0.23 ^a^	2.63 ± 0.15 ^b^	1.75 ± 0.04 ^c^
Histidine	-	0.71 ± 0.02 ^c^	2.88 ± 0.22 ^a^	3.56 ± 0.12 ^a^	1.70 ± 0.04 ^b^
Arginine	50.00	0.54 ± 0.01 ^d^	2.69 ± 0.14 ^a^	2.03 ± 0.11 ^b^	1.36 ± 0.03 ^c^
Total		4.13 ± 0.12 ^d^	22.73 ± 0.84 ^a^	16.02 ± 0.24 ^b^	10.03 ± 0.21 ^c^
Tastless					
Lysine *	20.00	0.37 ± 0.02 ^b^	0.81 ± 0.06 ^a^	0.71 ± 0.07 ^a^	0.48 ± 0.02 ^b^
Tyrosine	-	0.61 ± 0.02 ^d^	2.77 ± 0.23 ^a^	2.07 ± 0.19 ^b^	1.38 ± 0.03 ^c^
Cystine	-	0.02 ± 0.01 ^b^	0.01 ± 0.00 ^a^	0.05 ± 0.00 ^b^	0.03 ± 0.01 ^b^
Total		1.00 ± 0.05 ^d^	3.58 ± 0.29 ^a^	2.83 ± 0.23 ^b^	1.89 ± 0.05 ^c^
TEAA		3.06 ± 0.11 ^d^	16.62 ± 0.44 ^a^	10.35 ± 0.30 ^b^	6.92 ± 0.16 ^c^
Total amino acids		10.60 ± 0.31 ^d^	47.46 ± 1.02 ^a^	41.94 ± 0.34 ^b^	26.35 ± 0.61 ^c^

Note: CK, untreated control; FSIH, Flavourzyme-treated hydrolysate; PSIH, Protamex-treated hydrolysate; BSIH, Bromelain-treated hydrolysate. TEAA represents the total essential amino acids. “*” indicates essential amino acids. Taste thresholds were obtained from Ref. [20]. “-” indicates no threshold or undetected, letters between different groups indicate significant differences (*p* < 0.05).

**Table 2:** 5′-nucleotides content (mg/g) of SIHs and EUC.

	CK	FSIH	PSIH	BSIH
5′-CMP	0.15 ± 0.01 ^d^	0.28 ± 0.00 ^a^	0.25 ± 0.01 ^c^	0.26 ± 0.00 ^b^
5′-AMP	0.03 ± 0.00 ^a^	0.05 ± 0.01 ^bc^	0.07 ± 0.00 ^c^	0.04 ± 0.00 ^b^
5′-UMP	0.48 ± 0.01 ^d^	0.72 ± 0.00 ^a^	0.63 ± 0.00 ^b^	0.60 ± 0.00 ^c^
5′-GMP	0.45 ± 0.00 ^c^	0.58 ± 0.00 ^b^	0.59 ± 0.00 ^a^	0.58 ± 0.00 ^b^
5′-IMP	0.01 ± 0.00 ^a^	0.01 ± 0.00 ^a^	0.01 ± 0.00 ^a^	0.01 ± 0.00 ^a^
Total 5′-nucleotides	1.11 ± 0.01 ^d^	1.63 ± 0.01 ^a^	1.54 ± 0.00 ^b^	1.49 ± 0.01 ^c^
EUC (g MSG/100 g)	27.73 ± 0.61 ^d^	102.99 ± 8.52 ^b^	115.81 ± 6.88 ^a^	76.36 ± 2.68 ^c^

Note: CK, untreated control; FSIH, Flavourzyme-treated hydrolysate; PSIH, Protamex-treated hydrolysate; BSIH, Bromelain-treated hydrolysate; EUC, equivalent umami concentration; Different letters within the same row indicate significant differences (*p* < 0.05).

## Data Availability

The original contributions presented in this study are included in the article/Supplementary Materials. Further inquiries can be directed to the corresponding authors.

## Supplementary Materials

The following supporting information can be downloaded at: https://www.mdpi.com/article/10.3390/foods15111924/s1. Table S1: TAV values of free amino acids in *S. imbricatus* hydrolysates under different protease treatments; Table S2: Relative peak areas of volatile compounds in SIHs under different protease treatments determined by HS-GC-IMS; Table S3: ROAV values of aroma-active compounds in *S. imbricatus* hydrolysates under different protease treatments.

## Abbreviations

The following abbreviations are used in this manuscript:

*S. imbricatus*	*Sarcodon imbricatus*
SIHs	*S. imbricatus* hydrolysates
FSIH	Flavourzyme *Sarcodon imbricatus* hydrolysates
BSIH	Bromelain *Sarcodon imbricatus* hydrolysates
PSIH	Protamex *Sarcodon imbricatus* hydrolysates
5′-GMP	5′-guanosine monophosphate
5′-IMP	5′-inosine monophosphate
5′-AMP	5′-adenosine monophosphate
5′-UMP	5′-uracil monophosphate
5′-CMP	5′-cytidine monophosphate
MSG	monosodium glutamate
EUC	Equivalent umami concentration
HS-GC-IMS	Headspace-gas chromatography-ion mobility spectrometry
VOCs	Volatile organic compounds
FAAs	Free amino acids
TAV	Taste Activity Value
DH	Degree Hydrolysis
VIP	Variable Importance in Projection
UAA	Umami amino acids
SAA	Sweet amino acids
BAA	Bitter amino acids
NAA	Non-taste amino acids
TAA	Total amino acids
ETAA	Essential amino acids
OPLS-DA	Orthogonal partial least squares-discriminant analysis
PCA	Principal component analysis
