# Supplementary material for: Comparative Effects of Different Proteases on the Non-Volatile Taste Compounds and Volatile Flavor Fingerprints of Sarcodon imbricatus Hydrolysates
Source: Foods. 2026 May 29;15(11):1924. doi: 10.3390/foods15111924 (PMC13256879; doi:10.3390/foods15111924)
Supplement: Supplementary file 1 [file foods-15-01924-s001.zip › foods-4321499-supplementary.pdf]

Table S1 TAV values of free amino acids in *S. imbricatus* hydrolysates under different protease treatments

Free amino acids	Free amino acids taste threshold(mg/100g	TAV			
		CK	FSIH	PSIH	BSIH
Aspartic acid(Asp)	100	0.74±0.02[c]	2.03±0.17[a]	2.33±0.20[a]	1.56±0.03[b]
Glutamic acid(Glu)	30	6.96±0.15[c]	20.31±1.76[a]	22.35±1.43[a]	15.00±0.49[b]
Threonine*(Thr)	260	0.20±0.00[a]	0.85±0.04[c]	0.70±0.05[c]	0.47±0.01[b]
Serine(Ser)	150	0.30±0.02[c]	1.15±0.06[a]	1.06±0.09[a]	0.70±0.02[b]
Glycine(Gly)	130	0.11±0.01[d]	0.46±0.01[a]	0.39±0.02[b]	0.26±0.01[c]
Alanine(Ala)	60	2.33±0.07[c]	7.56±0.53[a]	7.62±0.52[a]	5.10±0.11[b]
Valine*(Val)	40	1.95±0.08[d]	10.57±0.73[a]	6.71±0.47[b]	4.48±0.09[c]
Methionine*(Met)	30	0.17±0.04[c]	0.91±0.12[a]	0.74±0.06[a]	0.50±0.03[b]
Isoleucine*(Ile)	90	0.55±0.02[d]	3.88±0.19[a]	1.93±0.11[b]	1.30±0.03[c]
Leucine*(Leu)	190	0.45±0.02[d]	2.96±0.29[a]	1.66±0.11[b]	1.11±0.02[c]
Phenylalanine*(Phe)	90	0.78±0.02[d]	3.94±0.25[a]	2.92±0.17[b]	1.94±0.04[c]
Arginine(Arg)	50	1.08±0.02[d]	5.37±0.28[a]	4.05±0.23[b]	2.72±0.05[c]
Lysine*(Lys)	20	1.85±0.10[b]	4.05±0.32[a]	3.57±0.35[a]	2.38±0.10[b]

Note: Different uppercase and lowercase letters indicate significant differences among groups ($p < 0.05$). The same applies below.

Table S2 Relative peak areas of volatile compounds in SIHs under different protease treatments determined by HS-GC-IMS

Compounds	Odor description	CAS	Formula	RI	Rt [sec]	Dt [a.u.]	Relative peak areas			
							CK	FSIH	PSIH	BSIH
Alcohols										
1-Butanol,3-methyl-	whiskey, malt, burnt	123-51-3	C5H12O	1210.6	743.742	1.24756	1452.38±546.79[b]	3864.72±70.11[a]	2875.50±665.89[a]	4033.69±41.94[a]
1-Pentanol-D	balsamic	71-41-0	C5H12O	1259.6	903.257	1.25428	351.12±26.78[b]	464.44±20.08[a]	469.37±60.50[a]	515.92±25.53[a]
1-Propanol-M	alcohol, pungent	71-23-8	C3H8O	1040.9	397.961	1.11004	1429.18±419.11[a]	661.45±46.08[c]	1130.46±5.23[ab]	858.21±59.96[b]

2- butanol-D	wine	78-92-2	C4H10O	1011.4	361.931	1.33866	301.76±25.29[b]	436.70±7.95[a]	459.28±6.39[a]	333.24±16.75[b]
2- butanol-M	wine	78-92-2	C4H10O	1011.8	362.409	1.13723	164.97±23.66[a]	144.16±4.25[a]	149.10±3.60[a]	155.84±8.47[a]
2-Heptanol	mushroom	543-49-7	C7H16O	1321.5	1097.743	1.36459	74.31±1.59[b]	63.69±3.08[c]	98.52±5.70[a]	77.13±2.53[b]
2-Methyl-2-propanol	camphor	75-65-0	C4H10O	912	294.879	1.31851	1104.12±426.44[a]	1280.05±171.64[a]	1043.35±326.25[a]	1000.59±400.99[a]
2-octanol		4128-31-8	C8H18O	1002.1	350.652	1.45643	992.06±47.54[b]	1445.25±20.42[a]	1495.50±18.41[a]	1080.45±61.66[b]
3-Furanmethanol		4412-91-3	C5H6O2	975.7	331.515	1.11259	275.07±4.69[a]	218.33±4.28[b]	236.14±6.04[ab]	215.66±16.36[b]
4-methyl-2-pentanol	pungent alcohol	108-11-2	C6H14O	1186.9	671.583	1.55319	540.64±192.77[ab]	556.75±83.46[ab]	747.42±96.92[a]	409.13±34.57[b]
Esters										
Ethyl formate-M	pungent	109-94-4	C3H6O2	831.9	248.866	1.06976	440.82±136.02[a]	404.95±113.91[a]	443.99±55.92[a]	478.25±63.87[a]
Ethyl formate-D	pungent	109-94-4	C3H6O2	835.3	250.844	1.20673	221.00±38.28[a]	219.61±38.21[a]	237.52±22.05[a]	228.02±21.18[a]
Butyl formate	fruity plum rum brandy	592-84-7	C5H10O2	1002.4	350.945	1.50858	1354.42±56.46[b]	1387.36±13.36[b]	1260.26±53.45[b]	1672.50±72.02[a]
2-methyl-1-propyl acetate	fruit, apple, banana	110-19-0	C6H12O2	1022.1	375.003	1.23382	112.91±4.39[b]	133.23±5.73[b]	211.70±39.42[a]	225.60±37.21[a]
1-Butanol,3-methyl-,acetate	banana	123-92-2	C7H14O2	1130.1	541.965	1.29804	84.20±9.98[c]	542.81±2.89[a]	104.83±9.14[b]	103.84±2.29[b]
ethyl hex-3-enoate	fruity green tart pineapple tropical winey grape quince	2396-83-0	C8H14O2	1325.3	1109.176	1.25642	383.16±32.53[b]	404.14±28.81[ab]	481.32±47.87[a]	390.25±24.54[ab]
Hexanoic acid propyl ester	furit	626-77-7	C9H18O2	1307	1054.278	1.38696	758.96±455.89[a]	848.47±140.01[a]	1027.62±221.09[a]	845.85±4.10[a]
hexyl formate	apple unripe plum banana sweet	629-33-4	C7H14O2	1363	1222.371	1.33189	219.66±64.09[b]	382.31±77.92[ab]	223.39±92.49[b]	541.43±41.89[a]
Isobutyl propanoate- M	fruity green ether sweet tutti frutti banana	540-42-1	C7H14O2	857	263.317	1.27791	411.20±27.18[c]	686.99±9.28[a]	616.59±27.83[a]	473.35±4.17[b]

(Z)-3-Hexenyl propionate	green pear apple fresh fruity waxy leafy	33467-74-2	C9H16O2	1101.6	476.803	1.38186	756.93±60.03[b]	970.05±34.85[a]	1179.72±53.70[a]	783.06±31.90[b]
(Z)-3-Hexenyl formate	fresh green waxy sweet vegetable grassy sharp	33467-73-1	C7H12O2	1258.9	900.873	1.71409	92.29±3.76[b]	110.58±24.04[b]	87.21±3.49[b]	190.88±16.03[a]
methyl 3-methylbutanoate	apple	556-24-1	C6H12O2	1013.2	364.167	1.52524	218.19±22.15[b]	357.66±24.71[a]	310.85±3.07[a]	307.65±3.30[a]
sec-butyl acetate	solvent fruity banana	105-46-4	C6H12O2	1002.6	351.238	1.21249	920.86±16.88[a]	559.50±32.76[b]	492.64±6.32[c]	827.91±27.60[a]
Butyl lactate	green fruity apple kiwi melon rind lactonic waxy winey apple skin soapy	138-22-7	C7H14O3	1013.3	364.3	1.27011	170.46±16.62[a]	173.49±2.85[a]	148.80±2.28[b]	183.88±6.73[a]
Methyl acetate	ether sweet fruity	79-20-9	C3H6O2	847.5	257.856	1.19425	3186.91±96.40[b]	3686.12±138.96[a]	3573.65±41.23[a]	3097.32±96.17[b]
phenylacetic acid butyl ester	rose honey chocolate	122-43-0	C12H16O2	1431.8	1428.536	1.44275	232.64±27.31[c]	747.18±27.70[a]	374.70±29.43[b]	269.19±24.67[c]
Octyl-acetate	rose honey chocolate	112-14-1	C10H20O2	1273.7	949.063	1.53901	131.70±15.86[a]	63.03±7.43[c]	125.00±2.40[a]	88.71±3.31[b]
Ketones										
1-Octen-3-one-M	herbal mushroom earthy musty dirty	4312-99-6	C8H14O	1306.9	1054.02	1.27244	2588.00±132.79[b]	3352.12±64.04[a]	3376.80±64.95[a]	2576.66±20.42[b]
1-Octen-3-one-D	herbal mushroom earthy musty dirty	4312-99-6	C8H14O	1306.4	1052.605	1.67928	471.36±17.75[c]	870.72±19.83[a]	949.32±37.43[a]	529.37±16.73[b]
2-Heptanone-M	soap	110-43-0	C7H14O	1183.2	663.164	1.26059	1109.50±85.15[b]	1501.15±80.38[a]	1443.94±16.88[a]	1449.14±25.70[a]
2-Heptanone-D	soap	110-43-0	C7H14O	1183.5	663.775	1.62658	318.87±81.28[c]	517.78±26.21[ab]	615.28±84.61[a]	336.49±31.37[bc]
2-methyl-2-hepten-6-one	pepper, mushroom, rubber	110-93-0	C8H14O	1341.3	1157.305	1.18292	776.27±211.70[b]	1207.39±64.87[a]	870.07±22.61[ab]	738.18±26.22[b]
3-Octanone	herb, butter, resin	106-68-3	C8H16O	1258.9	900.873	1.30832	287.16±70.92[b]	547.60±104.86[ab]	296.58±104.55[b]	895.58±81.60[a]

4-Methyl-2-pentanone	sharp solvent green herbal fruity dairy spice	108-10-1	C6H12O	1013.1	364.09	1.46943	153.30±3.30^{b}	279.44±4.17^{a}	276.78±2.35^{a}	164.06±11.63^{b}
Aldehydes										
(E)-2-nonenal-D	Cucumber, fat, green	18829-56-6	C9H16O	1459	1510.243	1.95178	679.05±51.72^{a}	610.67±60.86^{a}	715.42±33.58^{a}	745.37±43.07^{a}
(E)-2-nonenal-M	Cucumber, fat, green	18829-56-6	C9H16O	1459.4	1511.231	1.39477	1873.86±173.37^{c}	2081.65±36.87^{bc}	2553.67±134.08^{ab}	2828.77±73.14^{a}
(E)-2-Pentenal	strawberry, fruit, tomato	1576-87-0	C5H8O	1128.5	538.244	1.36862	83.58±6.19^{c}	212.18±4.99^{a}	116.21±13.65^{b}	80.17±4.77^{c}
1-hexanal-M	grass, tallow, fat	66-25-1	C6H12O	1096.9	466.333	1.55667	373.77±319.36^{a}	233.55±309.98^{a}	689.29±275.70^{a}	426.64±138.96^{a}
1-hexanal-D	grass, tallow, fat	66-25-1	C6H12O	1085.2	452.037	1.55706	1388.08±125.36^{c}	1686.87±27.37^{b}	1934.28±99.88^{ab}	2153.43±22.55^{a}
1-nonanal-M	fat, citrus, green	124-19-6	C9H18O	1397.8	1326.664	1.48022	8349.83±2581.08^{a}	6942.96±1752.69^{a}	7664.13±737.10^{a}	4242.72±355.52^{b}
1-nonanal-D	fat, citrus, green	124-19-6	C9H18O	1397.3	1325.173	1.9431	2561.42±2626.52^{a}	1468.23±1043.72^{a}	1652.39±371.08^{a}	339.43±70.69^{a}
1-octanal	fat, soap, lemon, green	124-13-0	C8H16O	1294.2	1015.834	1.41019	2860.04±1531.22^{ab}	2226.51±1016.86^{ab}	2905.67±965.47^{a}	1218.15±167.47^{b}
2-methyl-2-propenal	wild hyacinth foliage	78-85-3	C4H6O	890.5	282.575	1.22496	3167.85±109.57^{ab}	3015.46±107.68^{ab}	3400.10±116.21^{a}	2898.73±133.66^{b}
2-Methylbutanal	Cocoa, almond	96-17-3	C5H10O	909.2	293.294	1.16096	137.68±51.42^{a}	131.56±16.11^{a}	100.00±28.66^{a}	118.50±46.77^{a}
3-Methyl-2-butenal	sweet fruity pungent brown nutty almond cherry	107-86-8	C5H8O	1203.9	721.912	1.09181	318.02±18.65^{b}	353.33±6.84^{ab}	378.86±16.57^{a}	241.27±7.28^{c}
Butanal	pungent, green	123-72-8	C4H8O	880.9	277.042	1.10796	469.45±5.19^{c}	522.52±4.72^{a}	485.85±4.65^{b}	450.59±1.84^{d}
(Z)-4-heptenal	biscuit, cream	6728-31-0	C7H12O	1274.4	951.212	1.14806	6593.95±405.86^{a}	4455.47±198.34^{c}	6191.20±46.66^{a}	5198.58±10.39^{b}
Heptaldehyde-M	fat, citrus, rancid	111-71-7	C7H14O	1187.8	673.554	1.33893	3341.68±1136.07^{ab}	2769.46±503.05^{ab}	3363.57±379.33^{a}	2214.47±145.64^{b}
Heptaldehyde-D	fat, citrus, rancid	111-71-7	C7H14O	1186.5	670.791	1.69611	1055.34±959.84^{a}	603.99±296.66^{a}	1038.24±352.29^{a}	319.23±59.94^{a}
Acids										
2-Methylheptanoic acid	waxy green cheese sweaty butter milk	1188-02-9	C8H16O2	1143.6	572.835	1.41008	260.54±8.73^{b}	271.45±30.16^{b}	363.38±8.20^{a}	220.49±9.77^{c}
Acetic acid	sour	64-19-7	C2H4O2	1507.6	1655.835	1.05712	13304.09±2439.77^{a}	6339.86±483.43^{b}	3893.85±566.10^{c}	3102.05±288.21^{c}

Heterocyclic										
(2,6)-dimethylpyrazine	roasted nut, cocoa, roast beef	108-50-9	C6H8N2	1306.5	1052.825	1.53644	111.03±6.29^{c}	176.03±2.89^{ab}	193.75±7.14^{a}	144.44±20.38^{b}
2-Methylpyrazine	popcorn	109-08-0	C5H6N2	1273.5	948.415	1.07475	990.07±34.14^{a}	610.20±20.45^{d}	832.96±27.03^{b}	678.10±8.46^{c}
2-Propylpyrazine	green vegetable nutty	18138-03-9	C7H10N2	1435.7	1440.365	1.22071	508.48±67.68^{c}	3592.90±31.30^{a}	1758.80±1230.42^{ab}	837.47±52.49^{bc}
Coumaran		496-16-2	C8H8O	1101.6	476.879	1.09179	138.88±24.81^{a}	151.41±22.93^{a}	154.87±13.39^{a}	110.85±1.96^{b}
2-ethyl furan	sweet burnt earthy malty	3208-16-0	C6H8O	953.4	318.713	1.297	1458.02±73.70^{a}	1403.87±42.13^{a}	1501.83±24.02^{a}	1424.73±119.49^{a}
2-pentyl furan	green bean, butter	3777-69-3	C9H14O	1231.7	812.488	1.25117	1121.28±41.20^{b}	1647.71±217.24^{a}	1705.09±216.91^{a}	1394.61±55.51^{a}
2-Methylthiophene	sulfur	554-14-3	C5H6S	1095.7	464.796	1.01626	38.99±8.62^{a}	39.83±8.69^{a}	54.62±2.70^{a}	55.05±3.81^{a}
Tetrahydrofuran		109-99-9	C4H8O	868.3	269.797	1.05513	6059.20±76.71^{a}	5198.77±235.78^{b}	5199.00±133.36^{b}	5838.61±188.68^{a}
Hydrocarbons										
1-octene	gasoline	111-66-0	C8H16	831	248.318	1.45295	336.97±79.68^{a}	329.41±20.64^{a}	335.93±40.66^{a}	307.31±19.12^{a}
(R)-alpha-pinene		14575-92-9	C10H16	937.7	309.68	1.28276	1328.32±111.37^{ab}	951.65±43.80^{b}	655.70±11.26^{c}	1774.26±421.80^{a}
alpha-Pinene		2437-95-8	C10H16	1037	393.223	1.21093	275.04±32.78^{c}	1289.40±61.77^{a}	435.01±13.54^{b}	258.26±13.80^{c}
2,2,4,6,6-Pentamethylheptane		13475-82-6	C12H26	987.3	338.2	1.33866	681.22±20.66^{bc}	796.66±41.14^{ab}	631.33±32.05^{c}	813.78±38.14^{a}
Benzene, propyl-		103-65-1	C9H12	1184.4	665.993	1.51713	405.91±63.65^{c}	555.96±11.90^{ab}	624.28±49.67^{a}	418.50±12.55^{bc}
ethyl benzene		100-41-4	C8H10	1128.6	538.628	1.077	104.74±10.38^{b}	272.11±5.62^{a}	109.60±5.04^{b}	107.33±4.80^{b}
Others										
hexanenitrile		628-73-9	C6H11N	880.6	276.861	1.27964	279.19±1.48^{b}	600.10±10.94^{a}	573.59±23.49^{a}	297.12±17.42^{b}
1-propanethiol	cabbage natural gas sweet onion	107-03-9	C3H8S	877.4	275.017	1.17678	820.21±5.40^{c}	1033.06±16.09^{a}	1066.72±12.56^{a}	869.65±15.45^{b}
dimethyl sulfide	cabbage, sulfur,	75-18-3	C2H6S	822.9	243.702	0.95584	5580.82±950.23^{a}	5304.46±894.86^{a}	4769.49±488.89^{a}	5040.73±454.32^{a}

1,1-diethoxy ethane	gasoline fruit, cream	105-57-7	$C_6H_{14}O_2$	889.9	282.224	1.04156	448.32±9.53^{a}	345.34±8.43^{bc}	337.17±9.63^{c}	380.19±18.74^{b}

Table S3 ROAV values of aroma-active compounds in *S. imbricatus* hydrolysates under different protease treatments

Compounds	Threshold(mg/kg)	CAS	CK	FSIH	PSIH	BSIH
1-Octen-3-one	0.000003	4312-99-6	100.00	100.00	100.00	100.00
(Z)-4-heptenal	0.000025	6728-31-0	25.90	12.66	17.17	20.08
(E)-2-nonenal-D	0.00019	18829-56-6	1.31	1.01	1.19	1.82
dimethyl sulfide	0.00012	75-18-3	4.59	3.14	2.75	4.06
1-nonanal	0.0011	124-19-6	0.95	0.54	0.59	0.40
Heptaldehyde	0. 0028	111-71-7	0.15	0.09	0.11	0.09
